# An outbreak of HIV infection among people who inject drugs linked to injection of propofol in Taiwan

**DOI:** 10.1371/journal.pone.0210210

**Published:** 2019-02-08

**Authors:** Yu-Ching Huang, Yen-Fang Huang, Min-Hau Lin, Jyh-Yuan Yang, Yu-Hsin Liao, Hsiu-Yun Lo, Carl Latkin, Kenrad E. Nelson

**Affiliations:** 1 Centers for Disease Control, Ministry of Health and Welfare, Taipei, Taiwan; 2 Department of Health, Behavior and Society, Bloomberg School of Public Health, Johns Hopkins University, Baltimore, MD, United States of America; 3 Department of Epidemiology, Bloomberg School of Public Health, Johns Hopkins University, Baltimore, MD, United States of America; Centers for Disease Control and Prevention, UNITED STATES

## Abstract

**Introduction:**

The aim of this study was to report an HIV outbreak related to propofol-injection and the impact of regulating propofol on the HIV epidemic among people who inject drugs (PWID).

**Methods:**

A retrospective cohort study of 252 PWID who were diagnosed with an HIV infection between 2014 and 2017 in Taiwan. The propofol information was collected by routine epidemic surveillance and interviews. We linked several national databases to collect other related factors, including methadone maintenance treatment (MMT) attendance and incarceration. The serums were tested for recent infection by the LAg‐avidity EIA assay and relationship of the trains by the Phylogenetic tree analysis. Analyses were conducted using the R Surveillance package for retrospective modeling for outbreak detection. A multiple logistic regression was used to evaluate the association between propofol-injection and other related factors.

**Results:**

There were 28 cases reported with propofol-injection, all of which were reported in Central Taiwan. A total of 11 (50%) cases among 22 propofol-injectors with serums were recent infections, which were higher than that 33 (23.4%) of non-propofol group. The phylogenetic tree indicated that 6 propofol-injectors were grouped together with the same cluster in circular. The HIV epidemic curve among PWID revealed an outbreak of 82 in 2015, which then decreased to 43 in 2016 after propofol began to be regulated as a Schedule 4 controlled drug in August 2015. In a multiple logistic regression, attendance at methadone clinics was associated with a significantly higher risk for propofol-injection (adjusted OR = 2.43, 95% CI = 0.98–5.98), and HIV reported in the year 2015 was associated with an increased risk of propofol-injection (adjusted OR = 4, 95% CI = 1.08–14.86).

**Conclusions:**

Our data indicate that the government regulation of propofol as a controlled drug strategy was associated with significant reduction in the spread of HIV among PWID.

## Introduction

The high prevalence of HIV among people who inject drugs (PWID) in many countries represents a global health challenge [1]. The injectable drugs related to HIV epidemics are frequently heroin or other opiates and cocaine depending on drug supplies, use pattern, and other factors. Heroin injection is the most common route of HIV transmission among PWID in Asia [2]. Several studies document that methadone maintenance treatment (MMT), can reduce HIV transmission and injection drug use [3–8]. Detoxification for withdrawal syndromes with long-term non-pharmacological drug treatment is another opioid substitution therapy. Although there is no direct evidence that detoxification can prevent HIV infection [9], opioid-dependent patients receive medically assisted treatment often MMT and detoxification interchangeably, due to the high dropout rate for MMT and the problem of withdrawal for opioids.

Anesthesia-assisted rapid opioid detoxification (AAROD), a technique wherein opioid antagonists are administered with heavy sedation or anesthesia, was developed during the 1980s in Western countries with the purpose of reducing the discomfort of withdrawal but is not recommended by recent review papers and medical guidelines [10]. Reviews concluded that AAROD carried a significantly serious risk of adverse events and offered no benefits for less severe withdrawal or prolonged maintenance treatment [11]. One recent study from the USA reported that AAROD was associated with death and severe adverse events. Intravenous administration of propofol is one of the anesthesia and sedation drugs used in the AAROD procedure [12].

Some case reports and surveys indicated propofol has the potential for abuse [13–16]. The majority of cases with propofol abuse problems were healthcare workers [13, 15, 17–20]. Recently, some reports showed that laypersons also had a problem with propofol misuse [21, 22]. The issue of the risks of propofol misuse was given increased attention in 2009 after Michael Jackson‘s death from a mixture of propofol and others drugs.

A major HIV epidemic among the injection drug-using population in Taiwan emerged in 2003. In response to this HIV epidemic, a comprehensive harm reduction program targeted PWID was implemented in 2005, including MMT since February 2006 [23]. Before 2006, only detoxification medical management for opioid-dependent patients was available but not AAROD. Several studies have indicated that the implementation of a comprehensive harm reduction program was followed by a significant reduction in the HIV incidence rate among PWID in Taiwan [24]. The annual number of HIV reported cases among PWID has decreased dramatically, totaling 2,427 in 2005, 1,860 in 2006, 751 in 2007, 393 in 2008, 183 in 2009, 119 in 2010, 117 in 2011, 84 in 2012, 50 in 2013, and 54 in 2014. However, the decreasing trend in annually reported HIV cases among PWID was reversed in 2015 (the case number was 82). Since 2014, a local health bureau in Central Taiwan has been investigating cases of propofol injection through illegal routes. Since 2015, the Taiwan government has regulated propofol as a controlled drug. Here, we report on an assessment between propofol injection and HIV infection, the impact of regulating propofol on the HIV epidemic and the risk factors associated with propofol injection in PWID with HIV infection.

## Materials and methods

### Ethics statement

In Taiwan, serum samples and information regarding the source of infection or contacts were required to be collected by the HIV Infection Control and Patient Rights Protection Act. According to our national HIV Infection Control and Patient Rights Protection Act Article 12, it mentions that “The infected have the obligations to provide information regarding the sources of infection or contacts; Competent authorities may conduct investigations of the infected and their sources of infection or contacts.” Often public health nurses are the major interviewer to the infected for the investigations of the transmission sources of infection or contacts routinely. Information regarding risk behaviors related with HIV infected were required to be recorded into the National HIV/AIDS reporting and case management system. Our study used this database to link other data systems, and all data were fully anonymized prior to the analysis. In addition, this research protocol was approved by the Taiwan Centers for Disease Control Institutional Review Board Committee. And the IRB committee also waived the need of consent of all participants (No: IRB-106302 and IRB-106117).

### Study sample and sources of data

The study sample in this study consists of records from 252 PWID who were diagnosed with HIV infection and reported to Taiwan Centers for Disease Control between January 2014 and December 2017. To understand whether or not the study population used propofol, we asked participants about their past injection drug use history through routine epidemic surveillance. In addition, we also conducted extensive face-to-face interviews with 10 patients in order to understand the purchasing of propofol and the injection process when we found the propofol injection among PWID to help setting up future prevention strategy by the law (S1 and S2 Files).

In our study, we used a unique ID to link individuals to three data systems for our analysis: the National Correctional database of the Ministry of Justice, the National HIV/AIDS reporting and case management system and the National MMT system. The participants’ attendance at MMT was obtained from the National MMT system. All MMT clinics and hospitals provided information for all of the MMT patients. On the other hand, the Ministry of Justice provided the incarceration data for our cohort throughout the follow-up period. Personal identifiers were removed after the link was completed and prior to the analysis.

### Laboratory measurements

In our subjects, the HIV status of each individual was assessed by the Sedia HIV-1 LAg-Avidity EIA (Sedia Biosciences Corporation, Oregon, USA) to distinguish between recent or long-term infections. The principle of the test is based on the fact that in response to HIV-1 infection, the immune system induced low avidity HIV-1 antibodies early in the infection, and as time progresses, the immune system matures and produces high avidity HIV-1 antibodies [25].

Normalized OD (ODn) values were calculated for each specimen using a CAL specimen tested on the same plate as follows: ODn = specimen OD/median CAL OD. All controls and CAL specimens were tested in triplicate, diluted individually, and median values were determined. The recent/long term HIV-1 status is judged if the ODn of a specimen is < 1.5, then the specimen is considered as a recent infection. If the ODn is > 1.5 then the specimen is considered as a long-term infection.

HIV nucleotide sequences alignment was done by CLUSTAL W. The evolutionary history was inferred using the General Time Reversible model, the Best-Fit Substitution Model of Maximum Likelihood method. The tree is drawn to scale, with branch lengths reflecting the number of substitutions per site. The analysis involved 90 HIV-1 (+) nucleotide sequences. There was a total of 413 nucleotides in the final dataset. Evolutionary analyses were conducted using MEGA6. Bootstrap values were derived from 1000 replicates.

### Statistical analysis

All participants were divided into two groups according to their use of propofol. The difference in the demographic and other related factors were tested using a chi-square test. And using content analysis to interpret the interview data.

To evaluate whether an outbreak of HIV infection among PWID was due to propofol injection, we used the R package surveillance for outbreak detection in routinely collected surveillance data [26, 27]. The R-package surveillance provides retrospective modeling aberration detection in the resulting surveillance time series. In this package containing several surveillance algorithms to detect aberration, a commonly used method is the Farrington algorithm [28]. However, considering that our data are known to be subject to external influences, we used the Bayesian approach that constitutes a seamless framework for performing both estimation and subsequent prediction [29]. The Bayesian algorithm computes the threshold as the upper prediction interval boundary for a new observation based on a generalized additive model (GAM). Our model developed for PWID counts was used with the data of previous 12 months as reference values to generate a predictive distribution. The procedure works by comparing the observed count in the current week with an expected number, which is calculated based on predictive posterior distribution. There is an outbreak detection if the current observation exceeds the upper threshold.

To assess the related factors associated with propofol injection, we conducted multiple logistic regression models. The adjusted models controlled for subject age, sex, education, incarceration, MMT attendance, and reported year. Analyses were conducted using SAS version 9.4 and R package.

## Results

### Description of the study population

A total of 252 individuals were included in the study, among whom 216(85.7%) were male, and 36 (14.3%) were female. The median age at the time of their HIV diagnosis date was 38 years (range: 20–74 years). The residences of the subjects in the study were distributed with approximately 31% in the northwest, 46% in middle-west, and 23% in southwest. A total of 130 (51.6%) respondents had attended a methadone clinic before their HIV diagnosis. A total of 94 (37.3%) respondents had been incarcerated (Table 1).

**Table 1:** Characteristics of people who inject drugs (PWID) enrolled in the study cohort (N = 252).

	Total		Propofol injection				p-value
			No		Yes		
	N	%	n	%	n	%	
**Sex**							0.5668
Female	36	14.3	33	14.7	3	10.7	
Male	216	85.7	191	85.3	25	89.3	
**Age group**							0.0588
20–34	47	18.7	42	18.8	5	17.9	
35–44	113	44.8	95	42.4	18	64.3	
≥45	92	36.5	87	38.8	5	17.9	
**Education**							0.6815
≤9 years	153	60.7	137	61.2	16	57.1	
>9 years	99	39.3	87	38.8	12	42.9	
**HIV reported year**							0.0122
2014	54	21.4	51	22.8	3	10.7	
2015	82	32.5	66	29.5	16	57.1	
2016	73	29.0	65	29.0	8	28.6	
2017	43	17.1	42	18.7	1	3.6	
Ever MMT*							0.0677
No	122	48.4	113	50.5	9	32.1	
Yes	130	51.6	111	49.5	19	67.9	
**Ever incarcerated**							0.5191
No	158	62.7	142	63.4	16	57.1	
Yes	94	37.3	82	36.6	12	42.9	
**Living Area**							<0.0001
North	78	31.0	78	34.8	0	0.0	
Central	116	46.0	88	39.3	28	100.0	
South	58	23.0	58	25.9	0	0.0	
**HIV status**							0.009
Long-term infection	119	73.0	108	76.6	11	50.0	
Recent infection	44	27.0	33	23.4	11	50.0	
No specimen	89	-	83	-	6	-	

* MMT: methadone maintenance therapy

A total of 163 sera were available from 252 HIV-positive injection drug users, among which 44 (27%) were recent infections. The recent infection rate of the propofol injection (50%) was higher than that of the non-propofol group (23.4%), as evaluated by a chi-square test (p = 0.009) (Table 1).

There were 28 cases reported with propofol-injection in the analysis. The number of the propofol injections from 2014 to 2017 were 3 in 2014, 16 in 2015, 8 in 2016, and 1 in 2017. The propofol-injection rate of the ever MMT group (67.9%) was marginally higher than that of the no MMT group (32.1%), as evaluated by a chi-square test (p = 0.06). By the area of residence, the propofol-injection cases all lived in Central Taiwan specifically (Table 1).

By linking to the National MMT system and the National Correctional database of the Ministry of Justice, we found that 22 propofol injectors (78.6%) had heroin injection history. Furthermore, according to the National HIV/AIDS reporting and cases management system, 16 propofol injectors (57.1%) reported were still with heroin injection behavior in previous year before HIV diagnosis.

### The assessment of propofol-injection on the HIV epidemic curve

**Fig 1:**
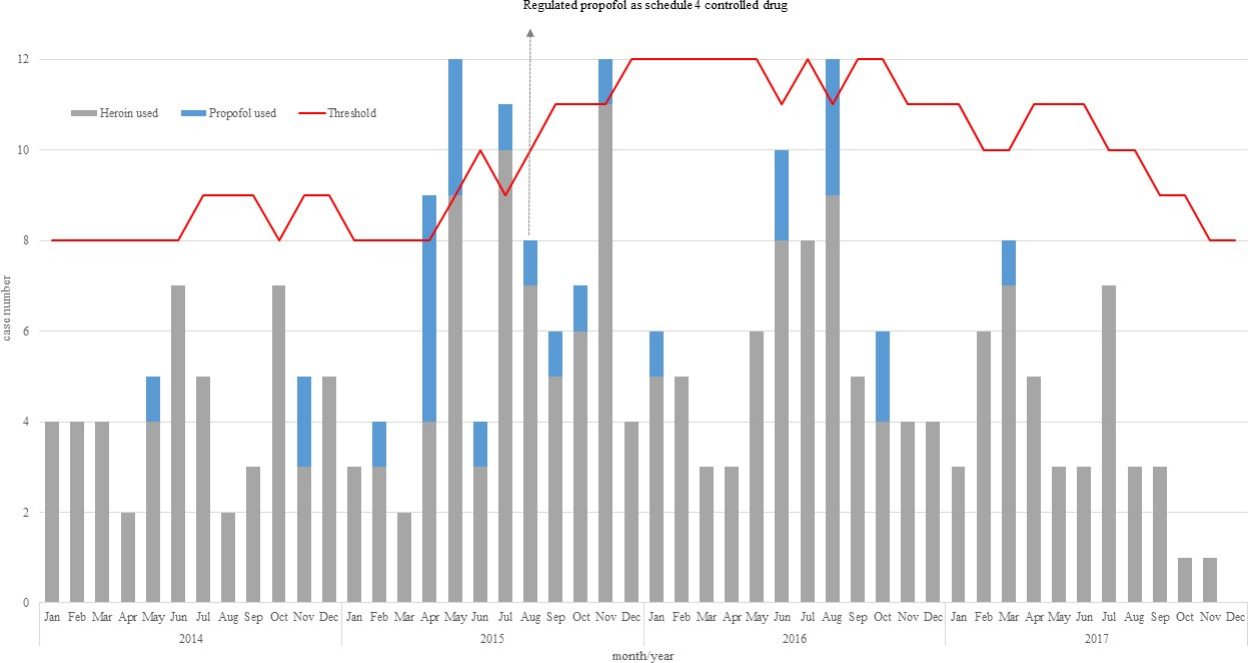
The temporal trend in PWID who were diagnosed HIV infection in 2014–2017 monitored by the Bayesian method.

Fig 1. shows the results of the monitoring with Bayesian method of the PWID who were diagnosed HIV infection by month from 2014 to 2017. In 2015, there were four months that the number of the newly reported HIV cases in PWID was out of the expected value, including April, May, July and November. The number of propofol injections was 1 and 2 in May and November 2014, respectively. However, the number of cases increased in 2015 with 1 in February, 5 in April, 3 in May, and 1 between June and November. Since 2016, the number had decreased, only 1 in January, 2 in June, 3 in August and 2 in October. And only one case was found throughout 2017. The HIV epidemic curve showed that the propofol injection in PWID contributed to the small outbreak.

### How to buy and inject propofol

There were two ways that PWID reported obtaining propofol. Some respondents obtained the propofol through specific community pharmacy stores between late 2013 and the first half of 2014. The pharmacists informed the patients that they provided a set with propofol for heroin addiction treatment when PWID bought needles and syringes in specific community pharmacy stores. The second channel was through the sellers that stood around MMT sites and slipped patients a small business card that had a simple sentence like “the new way to quit drugs” and a phone number written on it. The patients called the phone number and bought the propofol easily after leaving the MMT unit.

The respondents had a habit of injecting drugs with their friends in the same space, but they were separating injections by themselves. The respondents belonged to several different friendship groups. Within the same group, they knew each other’s HIV status and reporting that for a long time they had used clean needles and syringes for injecting heroin. Sometimes, they shared water due to a heroin shortage but had a sequential order of injecting in which the HIV positive individual injected last. When they started injecting propofol, they knew they needed to use clean butterfly needles for vein injections. However, the amount of propofol per bottle was 20 ml, and they could not finish the full volume before suddenly losing consciousness. Additionally, their butterfly needles sometimes were shedding. When they woke up and injected again, they could not distinguish the original owners of used butterfly needles. Some respondents told us that they shared the propofol bottle because they thought the long tubing of the butterfly needle could keep the propofol bottle clean.

### Phylogenetic tree analysis

**Fig 2:**
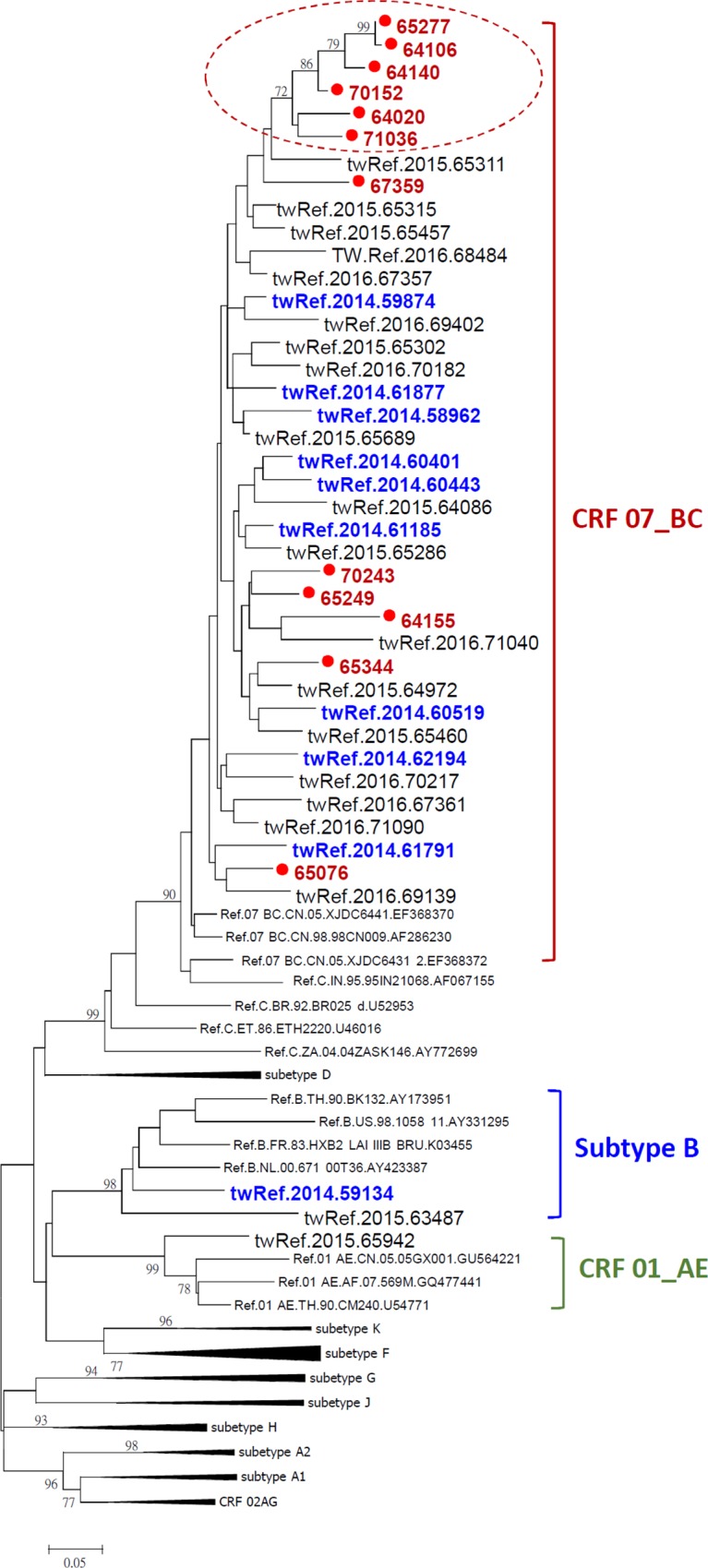
The result of phylogenetic tree analysis.

The phylogenetic analysis involved 90 HIV-1(+) nucleotide sequences including 12 HIV-1 (+) propofol-injection samples of this study, 10 Taiwanese HIV-1 strains in the same year and 68 HIV-1 reference strains. The nucleotide length of the 90 HIV-1(+) dataset is all trimmed to 413 nucleotide for comparison and phylogenetic analysis. Evolutionary analyses were conducted using MEGA6. Bootstrap values were derived from 1000 replicates. Based on the Maximum-likelihood phylogenetic tree indicating sequences relationships among Taiwan HIV strains and other reference strains, 6 propofol-injection samples were grouped together with the same cluster in circular. The other 6 propofol-injection samples were scatter around the HIV-1CRF07_BC and not group together in the same cluster in circular. (Fig 2) In addition, 67% clustered propofol-injection samples were long-term infection, in contrast to non-clustered samples (Table 2).

**Table 2:** The HIV status of 12 propofol injectors.

Variable	Total		Long-term infection		Recent infection	
	N	%	n	%	n	%
**Cluster**	6	50.0	4	67.0	2	33.0
**Others**	6	50.0	2	33.0	4	67.0

### Factors related with propofol injection

The multiple logistic regression models indicated that both MMT and HIV reporting year were associated with a significantly higher propofol injection rate among the sample (Table 3). The patients that had attended MMT clinics had a higher risk of propofol use (OR = 2.43, 95% CI = 0.98–5.98). We also observed that those who were diagnosed with HIV in the year 2015 were increased risk of propofol injection (OR = 4, 95% CI = 1.08–14.86). Gender, age, and education were not significantly associated with propofol injection.

**Table 3:** Factors associated with propofol injection from multiple logistic regression (N = 252).

	Adjusted OR	95% CI		P value
**Sex**				
Female	1	-	-	
Male	1.74	0.47	6.51	0.41
**Age group**				
20–34	1	-	-	
35–44	1.42	0.46	4.35	0.54
≥45	0.38	0.09	1.46	0.16
**Education**				
≤9 years	1	-	-	
>9 years	1.23	0.52	2.92	0.63
**HIV reported year**				
2014	1	-	-	
2015	4.00	1.08	14.86	0.04
2016	2.11	0.52	8.63	0.30
2017	0.38	0.04	3.89	0.42
Ever MMT*				
no	1	-	-	
yes	2.43	0.98	5.98	0.05
**Ever incarcerated**				
no	1	-	-	
yes	0.86	0.36	2.05	0.73

* MMT: methadone maintenance therapy

## Discussion

By analyzing data derived from the HIV reporting and case management system and interviews, we ascertained that propofol injection behavior affected HIV infection among PWID. This HIV outbreak involves a population PWID in the central area of Taiwan, where the HIV infection spread rapidly within social networks of people who injected propofol. In addition, the result of laboratory analysis indicated that propofol injectors had higher proportion of recent infection history. This result also supported our hypothesis of HIV outbreak during the study period. Past papers reported that AAROD with propofol was associated with deaths and severe adverse effects, such as cardiac necrosis and arrest, lethal hypoxemia, and myotoxic effects [16]. Our paper was the first study to report that previous propofol misuse or addiction was associated with HIV infection.

The HIV epidemic among PWID showed an outbreak in the central area of Taiwan in 2015 which then decreased. It was also found that the percent of propofol misuse in newly reported HIV cases decreased after 2015. A policy strategy was the key factor for reducing propofol misuse. In Taiwan, propofol for medical use was not restricted before 2015. In August 2015, the Taiwan Food and Drug Administration (TFDA) regulated propofol as a Schedule 4 controlled drug under the Controlled Substances Act in Taiwan due to several accidental deaths with propofol abuse. It was also reported that a medical doctor was arrested for promoting and selling propofol to PWID for opioid detoxification in Central Taiwan. The propofol as a Schedule 4 controlled drug indicates that pharmacies need to obtain permission from the TFDA to import or produce propofol. Then, monthly reports are prepared in accordance to the final retail sales objects' data of that drug; these reports are sent to the TFDA and to the local health administration at each sales location. When propofol became regulated as a controlled drug, it was found that the number of HIV reported cases in PWID with propofol injection was also decreased because the drugs became hard to obtain. Past studies revealed that the majority of propofol abusers were medical professionals [13, 15, 17–20]. The phenomenon was explained by theorizing that only medical professionals in hospitals or clinics had easy access to the drug. However, the possibility that some of them distributed the drug to the general population on purpose existed, the results suggest that it made severe health problems such as HIV infection spread quickly in Taiwan. On the other hand, when propofol was regulated as controlled drug, it was easy to block the abuse potential of propofol in the general population. Our results provide important evidence in support of regulating propofol as a controlled drug, which was recently initiated and rapidly expanded to control an emerging HIV/AIDS epidemic among PWID.

According to the result of phylogenetic tree analysis, 6 propofol-injectors were grouped together with the same cluster in circular. It indicated that the sharing behavior with propofol was existed. In the other site, there were 6 propofol injectors with no relationship. Our study population were all HIV new reported cases, but not all were HIV-positive with drug injection history. We speculated that some of the previous PWID with HIV infected also had propofol injection behaviors.

Individuals in our study were selected from a group with HIV infection, drug injection and unprotected risky injection behaviors. Only a portion of the cases in our study ever enrolled in MMT clinics, but the rate of propofol injection was significantly higher in those who had enrolled in an MMT group. Historically, some opiate users reported that they still injected heroin while they were under MMT because they preferred to experience the high. This finding suggests that patients in our study may have had higher levels of opiate use and still suffered from heroin addiction even while enrolled in MMT clinics. When patients heard about the new propofol treatment, they tried it definitely due to their limited information and drug dealers targeted this vulnerable group. For this reason, the study suggests that comprehensive drug education, such as avoiding inappropriate drug replacement for MMT patients, is needed.

Our analysis was also limited to the data available in the databases and the selected group with HIV infection. Thus, like other observational studies of surveillance, it must be noted that the population may not represent the behavior patterns of the entire PWID or MMT population. Despite this limitation, the strength of our study includes two ways of collecting information: surveillance system and case interviews.

In conclusion, our study found that propofol misuse was associated with HIV infection. The main channel for distribution of propofol to PWID was through medical professionals such as doctors or pharmacists. The government’s regulation of propofol as a controlled drug strategy in Taiwan has been associated with a substantial reduction in the spread of HIV among PWID and reduction in propofol use. Our findings suggest that countries with the potential for propofol abuse should consider national regulation through controlled drug status for prevention of HIV epidemics among PWID.

## Supporting information

S1 File Interview questionnaires. (DOCX)

S2 File Interview questionnaires (original language). (DOCX)
