# Supplementary material for: An outbreak of HIV infection among people who inject drugs linked to injection of propofol in Taiwan
Source: PLoS One. 2019 Feb 8;14(2):e0210210. doi: 10.1371/journal.pone.0210210 (PMC6368273; doi:10.1371/journal.pone.0210210)
Supplement: S1 File — (DOCX) [file pone.0210210.s001.docx]

**Interview questionnaires**

1. **Theme:** To understand the use of narcotic drugs (such as propofol, Thiamylal) and the situation of sharing needles among PWID.
2. **Interview object:** PWID with HIV.
3. **Interview questionnaires:**
4. **Topic 1: history of drug use**
5. When do you start using the drug? What drug do you use?
6. Have you ever used heroin? When did you start using it? How often do you use the drug? How did you use the drug?
7. Did you use multiple drugs at the same time? (likes heroin, propofol, or Thiamylal)
8. Have you ever used propofol? (if you answered “yes”, please answer the following questions)
9. How long did you use propofol? When did you start using it? How often do you use drugs? How do you use the drug?
10. Why did you use propofol?
11. How did you feel after using propofol?
12. When using propofol, was there anyone else around you? Do you think it is possible that the needle or drug used by others after falling asleep without knowing?
13. Have you ever shared needle, syringes or drugs with others? How often did you share?
14. Who did you use drug together in the past year? Besides these people, have any others used drug together?
15. **Topic 2: Harm reduction program**
16. Have you ever access drug abstention? How many times? What has been used for drug abstention?
17. How many times did you have been in prison? Why did you in prison?
18. Have you ever attend methadone maintenance treatment (MMT)? When is the first time you attend MMT? How many doses do you take? (highest and current dosage) Do you satisfy the hospital services? Is the dosage of methadone sufficient? Are you take methadone regularly?
19. Have you ever dropped out MMT? Why did you drop out?
20. How do you get the needle for injection in the past year?
21. Do you know needle and syringe program (NSP)? What experience in the use needle dispensing, needle recycling, and needle vending machines do you have?
