# Supplementary material for: An outbreak of HIV infection among people who inject drugs linked to injection of propofol in Taiwan
Source: PLoS One. 2019 Feb 8;14(2):e0210210. doi: 10.1371/journal.pone.0210210 (PMC6368273; doi:10.1371/journal.pone.0210210)
Supplement: S2 File — (DOCX) [file pone.0210210.s002.docx]

訪談問卷

1. 主題：了解愛滋藥癮感染者使用麻醉藥品(如：牛奶針、大象)型態及共用針具情形，進一步研擬防治對策。
2. 訪談對象：愛滋藥癮感染者。
3. 訪談問題：
4. 訪談主題一：個人用藥史
5. 什麼時候開始用藥？用什麼藥？
6. 海洛因用藥史？什麼時候開始使用？頻率？使用方式？
7. 有使用多種藥物嗎？（同時使用海洛因、牛奶針或大象？）
8. 是否曾經使用過牛奶針？(若有，續答5~9題)
9. 用牛奶針用多久？什麼時候開始使用？頻率？使用方式？
10. 為什麼會使用牛奶針？
11. 使用牛奶針後有什麼感受？
12. 使用牛奶針時，身邊有其他人嗎？有沒有可能睡著後針或藥被拿去使用而不知道？
13. 是否曾經和別人共用針具、稀釋液或是藥品？比率有多高？
14. 過去一年曾與誰一起打藥？除了那些人之外，還有曾跟其他人打藥嗎？
15. 訪談主題二：減害計畫使用情況
16. 是否曾戒癮過？幾次？用過什麼方式戒癮？
17. 入監次數？入監原因及情形？
18. 是否曾參加替代治療？第一次參加替代治療大約什麼時候？

劑量（最高和目前劑量）？醫院服務滿意度？美沙冬劑量是否足夠？服藥規則度？

1. 是否曾經中斷過替代治療有？為什麼？
2. 過去一年內，您注射用的針是如何取得的？
3. 是否知道清潔針具計畫？針具發放、針具回收、針具自動販賣機的使用經驗？
